# Supplementary material for: Association between oral microbiome and seven types of cancers in East Asian population: a two-sample Mendelian randomization analysis
Source: Front Mol Biosci. 2023 Nov 21;10:1327893. doi: 10.3389/fmolb.2023.1327893 (PMC10702768; doi:10.3389/fmolb.2023.1327893)
Supplement: Supplementary file 1 [file DataSheet1.ZIP › Supplementary Materials/MR plots of saliva/saliva═╝/ovarian cancer/pheno.2172_to_ovarian cancer_forest.pdf]

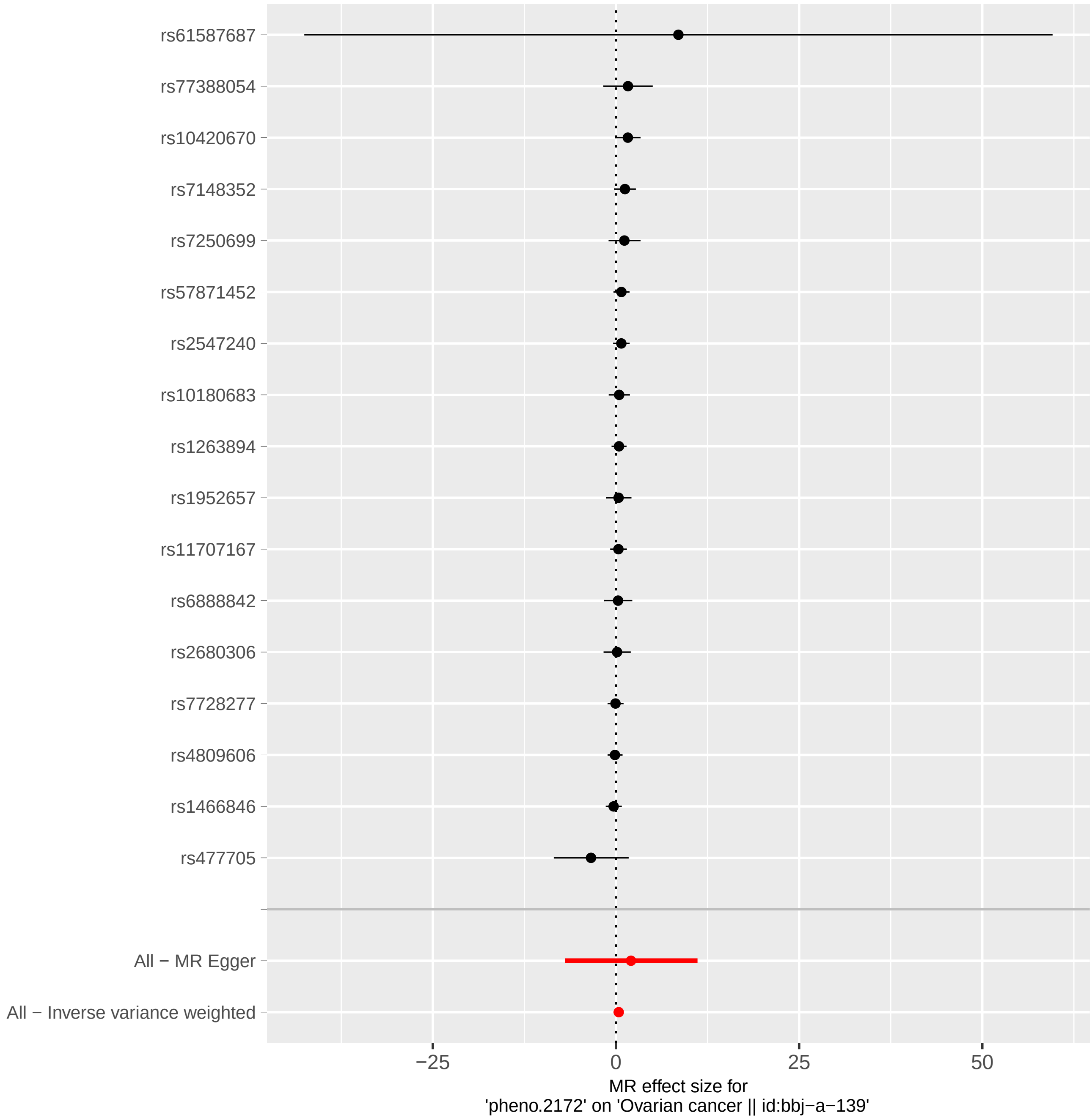
rs61587687
rs77388054
rs10420670
rs7148352
rs7250699
rs57871452
rs2547240
rs10180683
rs1263894
rs1952657
rs11707167
rs6888842
rs2680306
rs7728277
rs4809606
rs1466846
rs477705
All − MR Egger
All − Inverse variance weighted
−25
0
25
50
MR effect size for
'pheno.2172' on 'Ovarian cancer || id:bbj−a−139'
